# Augmented expression of TSPO after intracerebral hemorrhage: a role in inflammation?

**DOI:** 10.1186/s12974-016-0619-2

**Published:** 2016-06-17

**Authors:** Frederick Bonsack, Cargill H. Alleyne, Sangeetha Sukumari-Ramesh

**Affiliations:** Department of Neurosurgery, Medical College of Georgia, Augusta University, 1120 15th Street, CA1010, Augusta, GA 30912 USA

**Keywords:** Microglial activation, ICH, TSPO

## Abstract

**Background:**

Intracerebral hemorrhage (ICH) is a potentially fatal stroke subtype accounting for 10–15 % of all strokes. Despite neurosurgical intervention and supportive care, the 30-day mortality rate remains 30–50 % with ICH survivors frequently displaying neurological impairment and requiring long-term assisted care. Although accumulating evidence demonstrates the role of neuroinflammation in secondary brain injury and delayed fatality after ICH, the molecular regulators of neuroinflammation remain poorly defined after ICH.

**Methods:**

In the present study, ICH was induced in CD1 male mice by collagenase injection method and given the emerging role of TSPO (18-kDa translocator protein) in neuroinflammation, immunofluorescence staining of brain sections was performed to characterize the temporal expression pattern and cellular and subcellular localization of TSPO after ICH. Further, both genetic and pharmacological studies were employed to assess the functional role of TSPO in neuroinflammation.

**Results:**

The expression of TSPO was found to be increased in the peri-hematomal brain region 1 to 7 days post-injury, peaking on day 3 to day 5 in comparison to sham. Further, the TSPO expression was mostly observed in microglia/macrophages, the inflammatory cells of the central nervous system, suggesting an unexplored role of TSPO in neuroinflammatory responses after ICH. Further, the subcellular localization studies revealed prominent perinuclear expression of TSPO after ICH. Moreover, both genetic and pharmacological studies revealed a regulatory role of TSPO in the release of pro-inflammatory cytokines in a macrophage cell line, RAW 264.7.

**Conclusions:**

Altogether, the data suggest that TSPO induction after ICH could be an intrinsic mechanism to prevent an exacerbated inflammatory response and raise the possibility of targeting TSPO for the attenuation of secondary brain injury after ICH.

## Background

Intracerebral hemorrhage (ICH) is a common and often a fatal stroke subtype that accounts for 10–15 % of all stroke events [1]. In-hospital rates of mortality and patient disability following ICH are 40 and 80 % respectively [2] and the 1-month mortality rate is 30–50 % [3, 4]. In addition, a vast majority of ICH survivors do not regain their independence within 6 months of symptom onset and at 6 months only 20 % achieve independence in their daily lives [4, 5]. Despite recent advances in preclinical research, there is no effective treatment for ICH. Notably, the current treatment options are largely limited to providing mechanical ventilation and supportive care. The need for new therapeutic approaches for ICH has prompted a search for the molecular and cellular mechanisms that underlie brain damage after ICH.

TSPO is an 18-kDa trans-membrane protein of 169 amino acids, found primarily in the mitochondrial outer membrane [6]. TSPO was first identified as a diazepam-binding protein found in peripheral tissues; hence the previous denomination as peripheral-type benzodiazepine receptor [7, 8]. It is found in most species from bacteria to humans and the cDNA encoding TSPO has been cloned from various species such as rodents, bovines, and humans [9–13] among which there is an 80 % sequence homology [6]. TSPO has been implicated in many cellular processes including cell proliferation and differentiation, apoptosis, immunomodulation, tetrapyrrole biosynthesis, oxidative stress, steroid biosynthesis, and mitochondrial physiology [14–17]. In particular, TSPO was thought to be required and essential for the translocation of cholesterol from the outer mitochondrial membrane to the inner mitochondrial membrane, a limiting step for steroidogenesis [15, 18–20]. However, recent studies demonstrate that knockdown of TSPO in different cell types had no effect on viability and more importantly, that global TSPO knockout mice were viable and exhibited unaltered steroidogenesis [21–23], suggesting an elusive and conflicting role of TSPO in mammalian cells.

In the normal brain, TSPO expression is low, and it is found mainly in glia and at very low levels in neurons [8, 24–26]. However upon brain injury, augmented expression of TSPO is observed in activated glial cells [27–31] and serves as a biomarker for disease activity in Alzheimer’s and Parkinson’s disease [32, 33]. Synthetic ligands to TSPO are also investigated as therapeutic agents for various CNS disorders [34]. ICH results in both primary and secondary brain insults, and neuroinflammation characterized by glial activation is regarded as a major component of secondary brain injury mechanisms after ICH. However, the molecular expression and the functional role of TSPO after hemorrhagic brain injury have not been studied previously. The purpose of this study was to explore the temporal expression pattern and characterize the cellular localization of TSPO after ICH.

## Methods

### ICH

Animal studies were reviewed and approved by the Committee on Animal Use for Research and Education at Augusta University, in compliance with NIH and USDA guidelines. Male CD-1 mice (8–10 weeks old; Charles River) were anesthetized with an intraperitoneal injection of ketamine and xylazine and positioned prone in a stereotaxic head frame (Stoelting, WI, USA). A small animal temperature controller (David Kopf Instruments, USA) was used to maintain the body temperature at 37 ± 0.5 °C throughout surgery. With a high-speed dental drill (Dremel, USA), a 0.5-mm burr hole was made 2.2 mm lateral to the bregma, taking care not to damage the underlying dura. A 26-G Hamilton syringe containing 0.04 U of bacterial type IV collagenase (Sigma, St. Louis, MO, USA) in 0.5-μl saline was inserted with stereotaxic guidance 3.0 mm into the left striatum to induce spontaneous ICH [35, 36]. After removal of the needle, the burr hole was sealed with bone wax and the incision was surgically stapled. Sham animals underwent the same surgical procedure, but only a saline injection (0.5 μl) was performed. Mice were maintained at 37 °C until recovery.

### Immunohistochemistry

Deeply anesthetized mice were transcardially perfused with 0.1-M phosphate-buffered saline (pH 7.4; PBS), followed by 4 % paraformaldehyde in PBS. The brains were post-fixed overnight in 4 % paraformaldehyde and cryoprotected in 30 % sucrose at 4 °C until the brains were permeated. They were snap-frozen and sectioned at 25 μm using a cryostat. The coronal sections mounted on glass slides were incubated at 20 °C with 10 % normal donkey serum in PBS containing 0.4 % Triton X-100 for 1 h, followed by incubation with primary antibodies [Iba1 (ionized calcium binding adaptor molecule; 1:100; goat polyclonal; Abcam, MA, USA), TSPO (1:250; rabbit monoclonal; Abcam, MA, USA), glial fibrillary acidic protein (GFAP, 1:1000; goat polyclonal; Abcam, MA, USA), NeuN (1:100; mouse monoclonal; EMD Millipore, MA, USA), CD16/32 (1:100; rat monoclonal; BD Biosciences, CA, USA), CD206 (1:100; mouse monoclonal; BD Biosciences, CA, USA), and proliferating cell nuclear antigen (PCNA, 1:100; mouse monoclonal ; Cell Signaling, MA, USA)] at 4 °C for 24 h in 0.2 % Triton X-100 containing PBS. Sections were washed and incubated with appropriate Alexa Fluor-tagged secondary antibody (1:1000; Invitrogen/Life Technologies, USA; Alexa Fluor 594 conjugated donkey anti-Rat IgG, Alexa Fluor 594 or Alexa Fluor 488 conjugated donkey anti-Rabbit IgG, Alexa Fluor 594 or Alexa Fluor 488 conjugated donkey anti-Mouse IgG, and Alexa Fluor 488 conjugated donkey anti-Goat IgG) at room temperature for 1 h in 0.2 % Triton X-100 containing PBS. After washing, the sections were cover slipped with a mounting media containing DAPI (DAPI-Fluoromount-G; SouthernBiotech, AL, USA), a nuclear stain. Immunofluorescence was determined using a Zeiss LSM510 Meta confocal laser microscope and cellular colocalization was determined, as described earlier [37]. We analyzed 3 non-consecutive sections per animal and a minimum of 3–5 random fields around the hematoma. The fluorescence intensity of immunoreactivity and the number of immunopositive cells were estimated using ImageJ software (NIH, USA). The number of immunoreactive cells per mouse were quantified (3–5 fields per section and 3 sections per mouse, *n* = 3 mice/group) and averaged as positive cells per 0.1 mm^2^ in the peri-hematomal brain region. Quantitative assessment of colocalization between TSPO and CD16/32 or CD206 fluorescent signals was performed by calculating the overlap coefficient (ranging from 0 %; minimum colocalization to 100 %; maximum colocalization) using the Zeiss Zen2009 (NY, USA) software. An average of 10–15 cells were analyzed per section (3 sections per mouse; *n* = 3 mice/group) and overlap coefficient was calculated.

### Cell culture and enzyme-linked immunoassay (ELISA)

The mouse macrophage cell line, RAW 264.7 purchased from ATCC (American Type Culture Collection), was maintained in Dulbecco’s modified Eagle’s medium supplemented with 5 % fetal bovine serum and 5 % bovine growth serum, 5 % CO_2_, and 100 % humidity. Cells were added to tissue culture plates, allowed to adhere overnight, and then stimulated with hemin (30 μM). TSPO agonist or antagonist was added 1 h prior to hemin addition, and the hemin treatment was conducted for 18 h in the presence of TSPO agonist or antagonist and the supernatant was collected. The release of pro-inflammatory cytokines such as (tumor necrosis factor-α) TNF-α and (interleukin-6) IL-6 into the supernatant was estimated using ELISA as per manufacturer’s instructions. (RayBiotech, Inc., Norcross, GA, USA). Briefly, pre-coated 96-well ELISA plates for different captured antibodies were incubated overnight at 4 °C with cell culture supernatant and different concentrations of standard protein, 100 μl per well. Unbound materials were washed out, and biotinylated respective anti-cytokine detection antibody was added to each well. The plates were incubated for 1 h at room temperature. After washing, 100 μl of streptavidin-HRP conjugate was added to the wells and incubation was continued for another 45 min at room temperature. After washing, color development was performed by incubation with substrate solution. After adding stop solution, the optical density at 450 nm was determined for each well using a microtiter plate reader (Bio-TeK, Epoch) and the concentrations of the samples were determined in comparison to the respective standard concentration curve.

### Genetic knockdown of TSPO

RAW 264.7 cells were transfected with either control siRNA (ON-TARGETplus Non-targeting Pool; GE Dharmacon) or TSPO siRNA (SMARTpool: ON-TARGETplus TSPO siRNA; GE Dharmacon). Briefly, the cells were plated overnight and transfected with control or TSPO siRNA using HiPerFect Transfection Reagent (QIAGEN) according to manufacturer’s instructions. Target gene knockdown was verified 48-h post-transfection by qRT-PCR. To accomplish this, total RNA was isolated (SV RNA Isolation System, Promega), and quantitative RT-PCR was performed on a Cepheid SmartCycler II using a SuperScript III Platinum SYBR Green One-Step RT-PCR kit (Invitrogen, Carlsbad, CA, USA). Primers were as follows: TSPO (5′AGAAACCCTCTTGGCATCCG3′(F), 5′ GCCATACCCCATGGCTGAATA 3′(R) and RPS3: (FP 5′-AATGAACCGAAGCACACCATA-3′; RP 5′-ATCAGAGAGTTGACCGCAGTT-3′). Product specificity was confirmed by melting curve analysis and gene expression levels were quantified using a cDNA standard curve. Data were normalized to *RPS3*, a housekeeping gene that was unaffected by the experimental conditions.

### Statistical analysis

The data were analyzed using one-way analysis of variance followed by Student-Newman-Keuls post hoc test and were expressed as mean ± SE. A *p* value of <0.05 was considered to be significant.

## Results

### Temporal expression pattern of TSPO after ICH

**Fig. 1:**
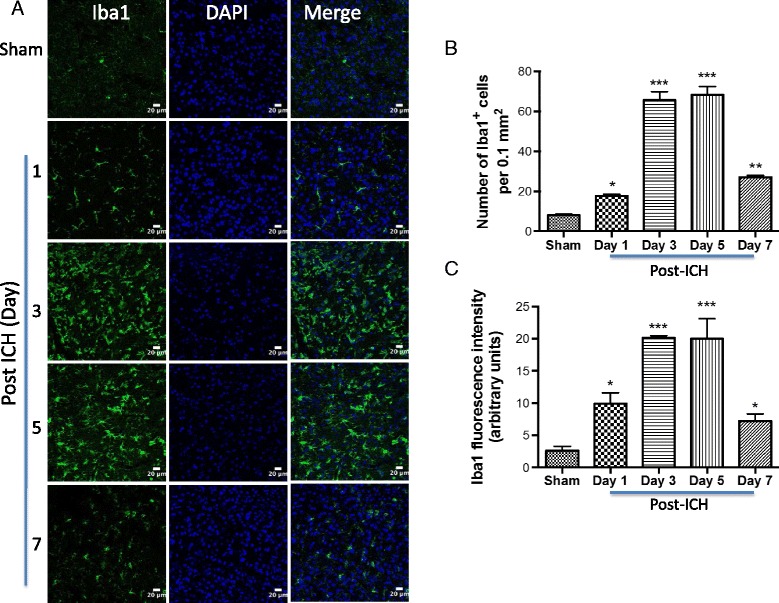
Microglial/macrophage activation and ICH. Brain sections were immunostained for Iba1 (**a**) as described in the “Methods” section. After counter staining the sections with DAPI, the staining was analyzed using confocal microscopy. ICH remarkably induced microglial/macrophage activation as evidenced by cellular hypertrophy with enhanced Iba1 staining. Scale bar = 20 μm *n* = 3–6/group. **b** The average number of Iba1-positive cells per 0.1 mm^2^ in the ipsilateral striatum. **c** The fluorescence intensity quantification of Iba1 immunoreactivity using ImageJ (NIH, USA). *p < 0.05, **p < 0.01, ***p < 0.001 vs. sham

**Fig. 2:**
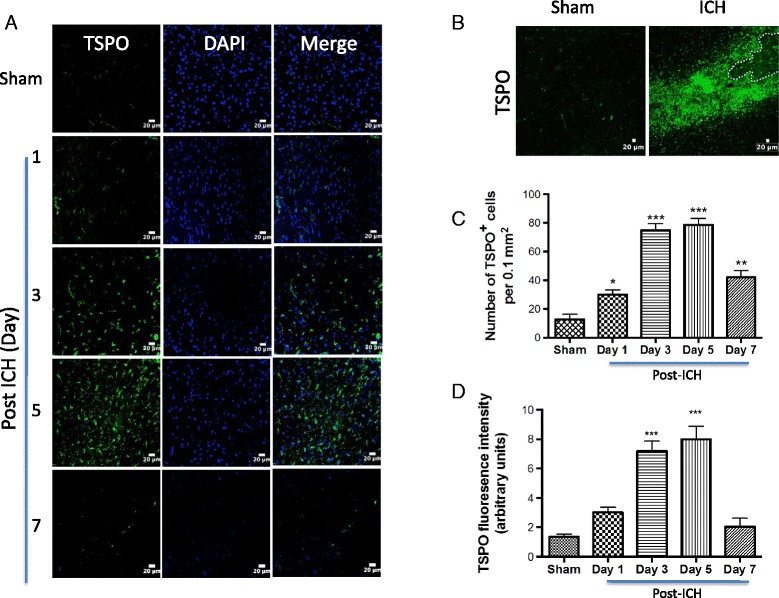
TSPO expression and ICH. **a** Representative confocal images demonstrating the temporal expression pattern of TSPO immunostaining in the brain tissue of ICH or sham. The confocal images were obtained from the peri-hematomal brain area of ICH 1–7 days post-injury or from the comparable brain region of sham. ICH remarkably augmented TSPO expression. Scale bar = 20 μm; *n* = 4–6/group. **b** The low magnification image depicting the profound induction of TSPO in the peri-hematomal brain region 5 days post-ICH or sham. **c** The average number of TSPO-positive cells per 0.1 mm^2^ in the ipsilateral striatum. **d** The fluorescence intensity quantification of TSPO immunoreactivity using ImageJ (NIH, USA). *p < 0.05, **p < 0.01, ***p < 0.001 vs. sham

Microglia are believed to be the first non-neuronal cells to react to a brain injury and are regarded as the major source of expression of pro-inflammatory cytokines after ICH [38]. Given the emerging role of TSPO in neuroinflammation, our goal was to characterize the immune response 1 to 7 days post-hemorrhagic injury because the kinetics of cytokine expression within the first week after experimental ICH is quite dynamic. In addition, important clinical sequelae begin to appear within the first 7-day post-ICH [39]. We found a very prominent microglia/macrophage activation at day 1 and it peaked at 3 to 5 days post-ICH as evidenced by a significant induction in both Iba1 (microglia/macrophage marker) immunofluorescence intensity and the number of Iba1-immunoreactive cells around the hematoma in comparison to sham (Fig. 1a–c). Notably, consistent with the previous reports [40, 41], Iba1-positive cells in the peri-hematomal area after ICH predominantly exhibited activated/reactive morphology with hypertrophic cell body and short processes in comparison to the ramified morphology observed in sham-injured mice (Fig. 1a). Notably, there was approximately an 8-fold increase (*p* < 0.001) in the number of Iba1-positive cells and Iba1 immunofluorescence intensity (*p* < 0.001) on day 3 and day 5, post-ICH in comparison to sham (Fig. 1b, c). However, it is known that Iba1 recognizes both microglia and macrophages, therefore the prominent induction in Iba1-positive cells after ICH could be either due to the proliferation and/or migration of microglia or due to the infiltration of macrophages after a brain injury. Furthermore, a week after ICH, microglia/macrophage activation tended to reduce; however, it did not reach sham levels (Fig 1a–c), suggesting a prolonged activation of microglia/macrophage after ICH. To evaluate TSPO expression after ICH, we performed immunofluorescence staining of brain sections acquired at different time points after surgery. TSPO was expressed at low levels in sham-operated mice as assessed by immunohistochemistry (Fig. 2) and no TSPO immunoreactivity was observed in the contralateral hemisphere (data not shown). In contrast, TSPO induction was observed in the peri-hematomal brain region 1 to 7 days post-injury with a remarkable up regulation observed on day 3 and day 5 post-injury (Fig. 2a, c, d). The quantification of the number of TSPO-immunoreactive cells and immunofluorescence intensity analysis together confirmed a significant induction of TSPO expression on day 3 and day 5 in comparison to sham (Fig. 2c, d). Along these lines, there was approximately a 6-fold increase (*p* < 0.001) in TSPO immunoreactivity on day 3 and day 5 post-ICH in comparison to sham (Fig. 2c, d).

### Cellular and subcellular localization of TSPO expression after ICH

**Fig. 3:**
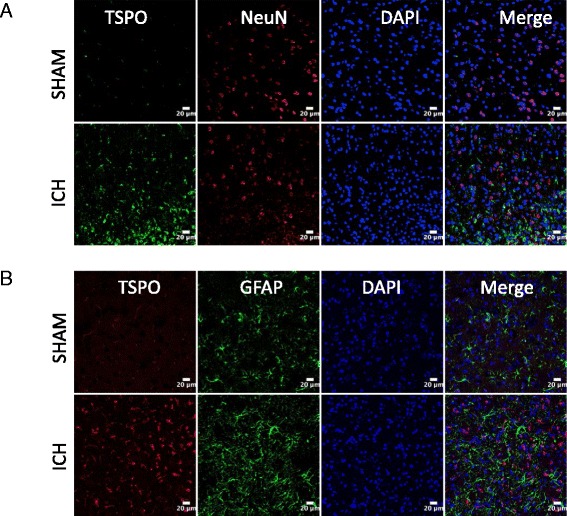
Cellular localization of TSPO. Double immunolabeling of brain sections from sham/ICH mice were performed for **a** TSPO and NeuN and **b** TSPO and GFAP 5 days post-surgery. TSPO expression was absent in both NeuN-positive and GFAP-positive cells after ICH. Scale bar = 20 μm; *n* = 3/group

**Fig. 4:**
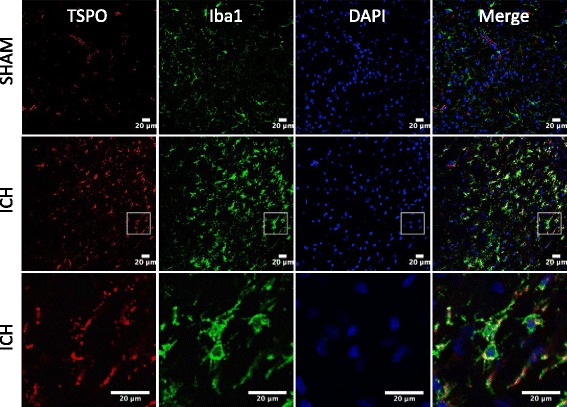
TSPO expression in microglia/macrophage. Double immunolabeling of brain sections were performed for TSPO and Iba1 5 days post-injury. A very prominent expression of TSPO was observed in Iba1-positive cells and the lowest panel depicts high magnification image. Scale bar = 20 μm; *n* = 3/group

**Fig. 5:**
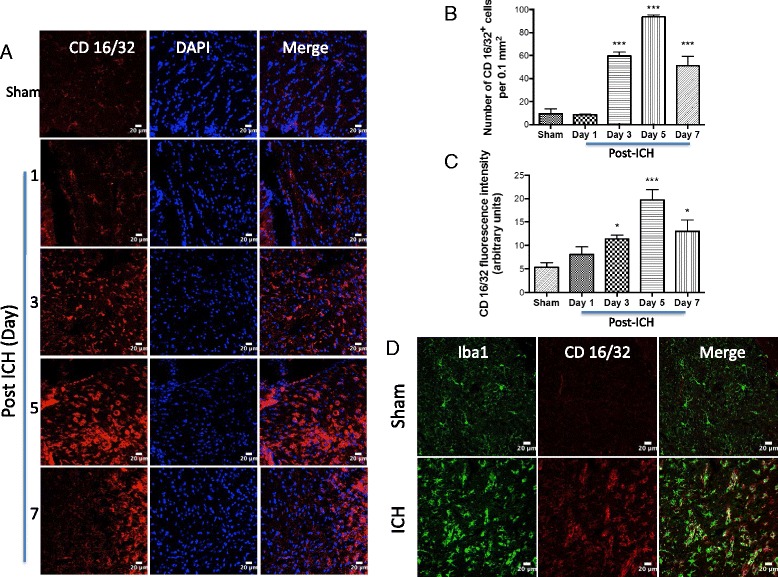
CD16/32 expression and ICH. **a** Immunofluorescent labeling was performed for CD16/32 on the brain sections from sham or 1–7 days post-ICH and was analyzed using confocal microscopy after counter staining the sections with DAPI. **b** The average number of CD16/32-positive cells per 0.1 mm^2^ in the ipsilateral striatum. **c** The fluorescence intensity quantification of CD16/32 immunoreactivity using ImageJ (NIH, USA). *p < 0.05, ***p < 0.001 vs. sham. **d** Brain sections from sham/ICH mice were immunolabeled for CD16/32 and Iba1, 5 days post-surgery. CD16/32 expression was mostly observed in Iba1-positive microglia/macrophage after ICH. Scale bar = 20 μm; *n* = 3–4/group

**Fig. 6:**
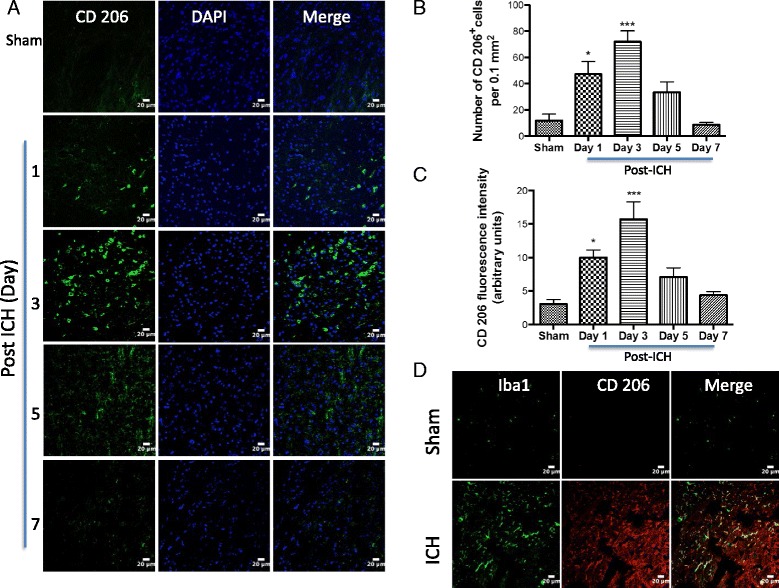
CD206 expression and ICH. **a** Brain sections were immunostained for CD206, as described in the “Methods” section. Staining was analyzed using confocal microscopy and images were obtained from the peri-hematomal brain tissue of ICH 1–7 days post-injury or from the comparable brain region of sham. **b** The average number of CD206-positive cells per 0.1 mm^2^ in the ipsilateral striatum. **c** The fluorescence intensity quantification of CD16/32 immunoreactivity using ImageJ (NIH, USA). *p < 0.05, ***p < 0.001 vs. sham. **d** Representative confocal images demonstrating the dual immunolabeling of brain sections for CD206 and Iba1 on day 3 post sham/ICH. Scale bar = 20 μm; *n* = 3–7/group

**Fig. 7:**
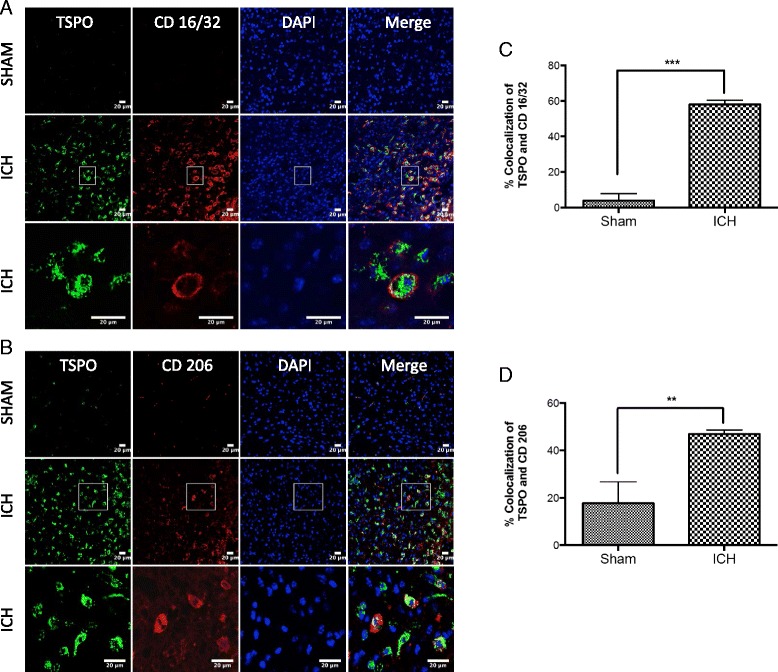
TSPO expression in M1 and M2 microglia/macrophage. Double immunolabeling of brain sections from sham/ICH mice were performed for **a** TSPO and CD 16/32 and **b** TSPO and CD206 on day 5 and day 3 post-surgery, respectively. The *lowest panel* depicts respective high magnification image. Scale bar = 20 μm. The percentage of colocalization between TSPO and CD16/32 (**c**) or CD206 (**d**) as assessed by calculating the overlap coefficient as described in the “Methods” section. **p < 0.01, ***p < 0.001 vs. sham

**Fig. 8:**
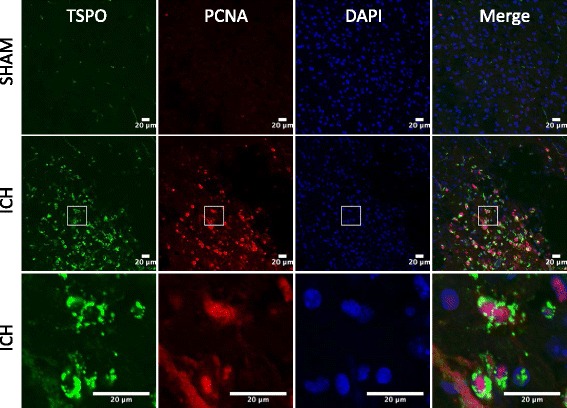
Subcellular localization of TSPO. Dual-label fluorescence immunohistochemistry was performed for proliferating cell nuclear antigen (PCNA), a cellular proliferation marker, and TSPO, in sham-operated mice or at 5 days post-ICH and prominent perinuclear expression of TSPO was observed after ICH and the *lowest panel* depicts high magnification image. Scale bar = 20 μm

To establish the cellular localization of TSPO after ICH, we employed double immunohistochemical labeling. TSPO expression was not found in GFAP- and NeuN-positive cells suggesting the absence of TSPO expression in astrocytes and neurons, respectively, after ICH (Fig. 3). In contrast, a remarkable colocalization of TSPO was observed in Iba1-positive cells, suggesting that TSPO-expressing cells after ICH are mostly microglia/macrophages, the inflammatory cells of CNS (Fig. 4). Notably, microglia/macrophages exhibited polarization after ICH and the analysis of immunopositive cell number and fluorescence intensity together revealed a significant induction of pro-inflammatory M1 microglia/macrophage marker, CD16/32 expression in the peri-hematomal brain region at 3 to 7 days post-injury, peaking on day 5, in comparison to sham (Fig. 5a–c). Further, CD16/32 expression was mostly confined to Iba1-positive cells after ICH (Fig. 5d) and the number of CD16/32-positive cells increased 6- and 10-fold (*p* < 0.001) on day 3 and day 5 post-ICH, respectively, in comparison to sham (Fig. 5b). Of note, the expression of anti-inflammatory M2 microglia/macrophage marker, CD206, was observed mostly on day 1 and day 3 post-ICH, with a peak expression observed on day 3 (Fig. 6a–c). There was a 6-fold increase (*p* < 0.001) in CD206-positive cells on day 3 post-ICH in comparison to sham (Fig. 6b) and the expression of CD206 colocalized with Iba1 (Fig. 6d). Moreover, both CD16/32 - and CD206-positive cells co-expressed TSPO further emphasizing the role of TSPO in neuroinflammatory responses after ICH (Fig. 7a, b). Quantitative colocalization analysis of images revealed a percentage of colocalization of 58.06 ± 2.3 and 46.97 ± 1.7 % between TSPO and CD16/32 or CD206, respectively, post-ICH (Fig. 7c, d). Though there was a difference in overlap of 11.09 % between the two, it was not statistically significant (data not shown). Furthermore, the subcellular localization of TSPO revealed prominent perinuclear expression of TSPO upon colocalization with PCNA, a nuclear marker of proliferating cells (Fig. 8).

### Pharmacological and genetic modulation of TSPO signaling significantly alters the release of pro-inflammatory cytokines

**Fig. 9:**
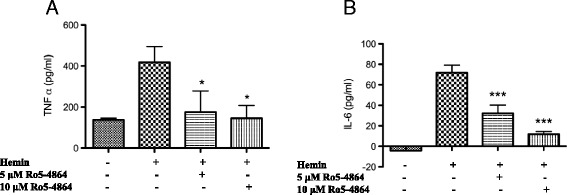
Ro5-4864-attenuated hemin-induced release of TNF-α and IL-6. RAW 264.7 cells were plated overnight and TSPO agonist, Ro5-4864, was added 1 h prior to hemin (30 μM) addition and the hemin treatment was conducted in the presence of Ro5-4864 and the supernatant was collected. The release of TNF-α (**a**) and IL-6 (**b**) into the supernatant was estimated using ELISA as per manufacturer’s instructions. (RayBiotech, Inc., Norcross, GA, USA). Ro5-4864 treatment significantly attenuated the hemin-induced release of TNF-α and IL-6. *n* = 4–9; *p < 0.05, ***p < 0.001 vs. hemin alone

**Fig. 10:**
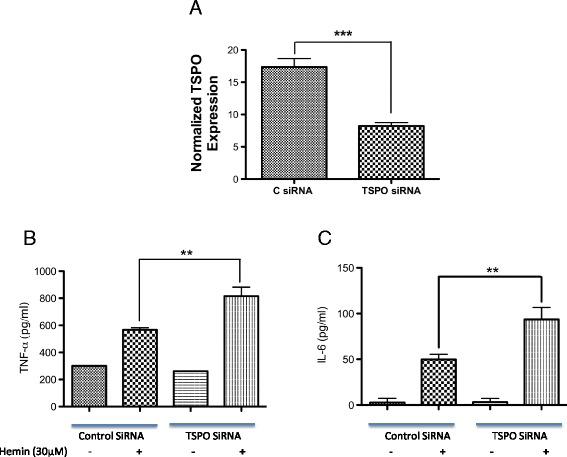
Genetic knockdown of TSPO augmented hemin-induced release of TNF-α and IL-6. **a** Genetic knockdown of TSPO in RAW. 264.7 cells were achieved employing TSPO siRNA as described in the “Methods” section. Genetic knockdown of TSPO significantly augmented the hemin-induced release of both TNF-α (**b**) and IL-6 (**c**) in RAW 264.7 cells. *n* = 3–4; **p < 0.01 vs. hemin-treated control, ***p < 0.001 vs. control

To establish the functional role of TSPO in microglial/macrophage activation, we studied whether a TSPO agonist, Ro5-4864, is able to modulate hemin-mediated inflammatory reaction. Hemin is a metabolite, which accumulates at high concentration in intracranial hematomas and is known to induce microglial activation via TLR-4 (Toll-like receptor-4) signaling mechanism [42]. Hemin treatment significantly augmented the release of pro-inflammatory cytokines TNF-α and IL-6 from murine macrophage cell line, RAW 264.7 cells, as evidenced by ELISA, whereas the incubation of cells with TSPO agonist, Ro5-4864, attenuated hemin-induced release of both TNF-α and IL-6 (Fig. 9). Along these lines, Ro5-4864 (5 and 10 μM) significantly reduced hemin-induced release of TNF-α by 87 and 97 %, respectively (Fig. 9a; *p* < 0.05 vs. hemin alone), and IL-6 by 52 and 79 %, respectively (Fig. 9b; *p* < 0.001 vs. hemin alone). Further, TSPO antagonist (PK11195) did not reduce hemin-induced TNF-α secretion (data not shown). To validate this further, we performed siRNA-mediated genetic knockdown of TSPO expression in RAW 264.7 cells and examined the inflammatory response. Notably, silencing of TSPO expression in RAW 264.7 cells by siRNA significantly reduced the TSPO expression by 52.6 % (Fig. 10a; *p* < 0.001 vs. control) and augmented hemin-induced release of TNF-α and IL-6 by 2-fold in comparison to controls (Fig. 10b, c; *p* < 0.01 vs. hemin-treated control). Altogether, the data derived from both pharmacological and genetic studies suggest that TSPO may serve as a negative regulator of inflammation after ICH.

## Discussion

Intracerebral hemorrhage (ICH) is a stroke subtype resulting from the spontaneous extravasation of blood into the brain parenchyma. Though chronic hypertension, amyloid angiopathy, and advanced age are recognized as the prominent risk factors of ICH, the risk factors also include but not limited to ethnic differences and lifestyle factors such as smoking and alcohol intake [43]. Currently, the worldwide incidence of ICH is 2 million cases per year [2], with approximately 120,000 cases per year in the USA [44–46]. However, the incidence is expected to have doubled by 2050 [39] due to aging and the spreading use of anticoagulants [47]. Though ICH is a major public health problem, no effective medical or surgical therapy has been firmly established. Given the critical role of microglia/macrophage-mediated inflammation in ICH-induced secondary brain injury, a thorough understanding of the molecular regulators of inflammation in hemorrhagic brain injury is critical. This study identifies for the first time the microglia/macrophage expression of TSPO and the existence of M1 and M2 microglia/macrophage phenotypes in a preclinical model of ICH.

Secondary inflammatory damage to brain tissue has emerged as a common link between different types of central nervous system disorders [48–51]. ICH results in both primary and secondary brain insults, and several clinical and preclinical studies have suggested a significant contribution of neuroinflammation to the pathophysiology of ICH [47, 52, 53]. Notably, activated microglia and newly recruited peripheral macrophages are regarded as the key cellular regulators of neuroinflammation after ICH based on their local release of cytokines, chemokines, prostaglandins, proteases, ferrous iron, and other immunoactive molecules [52, 54, 55]. Under normal conditions, microglia retain a relative quiescent phenotype for constant monitoring of the brain parenchyma [56]. As influenced by their environment, both resident microglia and peripheral macrophages assume pro-inflammatory M1 and anti-inflammatory M2 phenotypes. M1 polarized microglia/macrophages produce largely deleterious pro-inflammatory cytokines such as TNF-α, IL-1β, and pro-oxidant enzymes such as inducible nitric oxide synthase [57, 58]. In contrast, M2-polarized microglia/macrophages produce neurotrophic factors and have been associated with regenerative effects after brain injury [59]. However, the microglia/macrophage polarization after ICH is largely uncharacterized and herein we demonstrate for the first time the temporal pattern of microglia/macrophage polarization after ICH.

The inflammatory response to ICH is characterized by a rapid activation of resident microglia within minutes after the onset of ICH and the activated microglia/macrophage are found in and around the hematoma [52, 54, 60]. The early response of microglia/macrophage to ICH suggests that components of the hematoma may trigger microglial activation. Consistently, the hematoma components such as erythrocytes and its lysed products (Hb, heme, and iron), thrombin (blood coagulation factor), and complements are capable of activating microglia/macrophages [61–64]. However, the precise molecular mechanism of microglia/macrophage activation after ICH remains unclear. Further, hemin, a hemoglobin metabolite which accumulates at high concentration in intracranial hematomas [65], can induce inflammatory brain injury after ICH [64]. Altogether, inflammatory signaling triggered by hematoma components leads to microglial activation, peripheral inflammatory cell infiltration, and release of pro-inflammatory mediators, eventually resulting in massive brain cell death and neurological deficits. However, intrinsic regulators of microglia/macrophage activation after ICH remain largely uncharacterized.

Herein, we demonstrate for the first time that the TSPO expression is elevated after ICH and the peak expression is observed on day 3 to day 5 post-ICH, the time point that exhibits very prominent microglia/macrophage activation. Furthermore, TSPO expression was observed mostly in the Iba1-positive microglia or macrophage whereas TSPO expression was not observed in either GFAP- or NeuN-positive cells, making TSPO a suitable molecular target to modulate microglia/macrophage functions after ICH. Furthermore, microglia/macrophage exhibited both pro-inflammatory M1 and anti-inflammatory M2 phenotypes after ICH. The expression of M1 cell surface marker CD16/32 is found to be prolonged after ICH in contrast to an early occurrence of M2, cell surface marker, CD206 suggesting a possible role of pro-inflammatory M1 microglia/macrophage in ICH-mediated both acute and long-term neurological deficits. Further, the early occurrence of CD206-positive M2 microglia/macrophage after ICH could be an intrinsic mechanism to remove cellular debris derived from hematoma or to restore brain homeostasis. More importantly, the expression of TSPO in both M1 and M2 microglia/macrophage as demonstrated herein suggests an unexplored role of TSPO in neuroinflammatory responses after ICH. In addition, a TSPO agonist attenuated hemin-mediated release of TNF-α and IL-6 from RAW 264.7 cells, suggesting that TSPO can be therapeutically targeted to attenuate neuroinflammatory response after ICH and this was further supported by the genetic knockdown studies. However, further studies are required to characterize the cell-specific role of TSPO expression in M1 and M2 microglia/macrophage after ICH. Though, CD16/32 and CD 206 are widely used and regarded as signature markers of M1 and M2 microglia/macrophage phenotypes, respectively, the use of a single molecular marker in characterizing either M1 or M2 microglia/macrophage remains as a limitation of the study. Further, given the occurrence of differential microglia/macrophage phenotypes after a brain injury, the possibility of expression of TSPO in other microglia/macrophage phenotypes apart from M1 and M2 cannot be completely ruled out. Notably, LPS (lipopolysaccharide), a potent ligand of TLR-4 augmented TSPO expression in BV2 microglial cells [66]. Moreover, in actively proliferating cells and in LPS-induced cultured retinal microglia, the expression of TSPO has been observed in the perinuclear and nuclear area [66, 67]. Consistently, the subcellular localization of TSPO revealed prominent perinuclear expression after ICH suggesting a role of TSPO in microglial proliferation which is often associated with neurodegenerative conditions [68] further emphasizing the therapeutic potential of TSPO. It has been previously reported that IL-1β and TNF-α can stimulate microglial proliferation [69–71]. However, the mechanism by which TSPO regulates neuroinflammation after ICH needs further investigation.

Further, emerging evidences suggest that the administration of TSPO-selective ligands may be useful in the treatment of neuroinflammatory conditions [72–74] and may exert neuroprotection after seizures and brain injury by suppressing microglial activation [72, 75–78]. Moreover, minocycline-mediated attenuation of retinal degeneration was associated with reduction in microglial reactivity and the expression of TSPO [79]. Notably, the primary endogenous ligands of TSPO include a polypeptide called diazepam-binding inhibitor (DBI) and its shorter peptide cleavage products called endozepines [80]. These molecules are expressed and secreted by glial populations in the CNS [81]. It is postulated that the ligand-mediated TSPO signaling in microglia can increase mitochondrial cholesterol flux, facilitating the production of modulatory neurosteroids that in turn repress inflammatory genes in a cell-autonomous manner [66]. Endogenous TSPO signaling may also result in other physiological changes in microglia including regulation of calcium influx [82], mitochondrial function [6], and apoptosis [83], all of which may modulate microglial activation. However, neither the precise role of TSPO nor the exact mechanism by which TSPO-specific ligands confer neuroprotection is known. Though TSPO ligands have been widely used for brain imaging of neuroinflammation, in several neuropathological conditions such as Alzheimer’s disease (AD), Parkinson’s disease, and Huntington’s disease, no such imaging technique is currently available to monitor microglial activation after ICH. The low level of expression in uninjured brain regions coupled with the injury-induced remarkable induction makes TSPO a suitable candidate to monitor inflammation after ICH. Altogether, the data derived from this study suggest a therapeutic and diagnostic potential of TSPO after ICH.

## Conclusions

The present study reports for the first time that TSPO expression is found to be increased in the peri-hematomal brain region 1 to 7 days post-ICH, peaking on day 3 to day 5 in comparison to sham. Further, the expression of TSPO is mostly observed in microglia/macrophages suggesting a possible role of TSPO in neuroinflammatory response after ICH and this is further supported by its occurrence in M1 and M2 microglia/macrophage phenotypes after ICH. Notably, the subcellular localization studies revealed prominent perinuclear expression of TSPO after ICH. Though this observation is consistent with the in vitro studies with cultured microglia [66], the precise functional significance of TSPO expression in this subcellular area requires further investigation. Moreover, both genetic and pharmacological studies revealed a regulatory role of TSPO in the release of pro-inflammatory cytokines in a macrophage cell line, RAW 264.7. Altogether, the data suggest that TSPO induction after ICH could be an intrinsic mechanism to prevent an exacerbated inflammatory response and raise the possibility of targeting TSPO for the attenuation of secondary brain injury after ICH.

## Abbreviations

CD 16/32, cluster of differentiation 16/32; CD 206, cluster of differentiation 206; cDNA, complementary DNA; CNS, central nervous system; DAPI, 4′,6-diamidino-2-phenylindole; ELISA, enzyme-linked immunosorbent assay; GFAP, glial fibrillary acidic protein; Hb, hemoglobin; Iba1, ionized calcium binding adaptor molecule 1; ICH, intracerebral hemorrhage; IL-6, interleukin 6; LPS, lipopolysaccharide; NeuN, neuronal nuclei; PBS, phosphate-buffered saline; PCNA, proliferating cell nuclear antigen; RPS3, ribosomal protein S3; RT-PCR, reverse transcription polymerase chain reaction; SE, standard error; siRNA, small interfering ribonucleic acid; TLR-4, Toll-like receptor 4; TNF-α, tumor necrosis factor alpha; TSPO, 18-kDa translocator protein
